# Epidemiological aspects and spatial distribution of visceral leishmaniasis in Governador Valadares, Brazil, between 2008 and 2012

**DOI:** 10.1590/0037-8682-0216-2019

**Published:** 2019-12-20

**Authors:** Aimara da Costa Pinheiro, Alexandre Sylvio Vieira da Costa, Rodrigo Santos de Oliveira, Maria Letícia Costa Reis

**Affiliations:** 1 Secretaria Municipal de Saúde, Governador Valadares, MG, Brasil.; 2 Universidade Federal dos Vales do Jequitinhonha e Mucuri, Instituto de Ciência, Engenharia e Tecnologia, Teófilo Otoni, MG, Brasil.; 3 Universidade Vale do Rio Doce, Governador Valadares, MG, Brasil.; 4 Universidade Federal dos Vales do Jequitinhonha e Mucuri, Faculdade de Medicina, Diamantina, MG, Brasil.

**Keywords:** Visceral leishmaniasis, Spatial Analysis, Epidemiology

## Abstract

**INTRODUCTION:**

Visceral leishmaniasis (VL) is an important parasitic disease. We evaluated the epidemiological aspects and spatial distribution of visceral leishmaniasis in Governador Valadares, Brazil.

**METHODS:**

All cases of VL, registered by the municipal health department, were analyzed and georeferenced.

**RESULTS:**

The human mortality rate was 15% and canine seroprevalence rate was 29.0%. Higher numbers of canine VL cases correlated with higher incidence of human cases.

**CONCLUSIONS:**

The high rate of canine seroprevalence, resurgence of the human disease, and correlation between canine and human VL reinforces the role of the dog in disease transmission within the municipality.

Visceral leishmaniasis (VL) is a neglected and endemic parasitic disease reported in 65 countries. In 2017, 94% of the new cases occurred in seven countries: Ethiopia, India, Kenya, Somalia, South Sudan, Sudan and Brazil^1^. In Brazil, the etiological agent of VL is *Leishmania infantum*, which is widely distributed in the wild and domestic environments^2^. The main vector is the *Lutzomyia longipalpis* sandfly, and dogs (*Canis familiaris*) are the main urban reservoir in domestic and peridomicile environments, contributing to the life cycle of the disease in urban areas^3^.

Dogs are responsible for endemic and epidemic VL in large urban areas^4^. The canine enzootic disease preceded the occurrence of human cases, and the infection has been more prevalent in dogs than in humans^5^.

The first human cases of VL in Vale do Rio Doce, Minas Gerais (MG) were reported in 1966, in a predominantly rural area^6^. Since then, no further cases were reported and the disease was considered to be under control. In 2008, the disease re-emerged in Governador Valadares, considered an endemic area due to the active transmission of VL^7^. To date, no epidemiological studies of leishmaniasis using spatial analysis have been performed. Spatial analysis would enable studying the superposition of human and canine VL cases, and this may aid the planning of disease control measures.

Therefore, the present study evaluated the spatial distribution and epidemiological aspects of VL in the municipality of Governador Valadares, MG, between 2008 and 2012.

Governador Valadares (18º 51’ 2’’S, 41º 56’ 53’’W) is located in the eastern region of the state of MG, in the mesoregion of Vale do Rio Doce. In 2010, the demographic census estimated the population to be 263,689^8^.

This study included all individuals diagnosed with VL, confirmed by the clinic-laboratory criteria, registered in the urban area by the epidemiology administration (GEPI) of the Department of Health Surveillance, Municipal Health Secretariat of the city of Governador Valadares, from 2008 to 2012. The serology techniques included immunochromatographic rapid test and indirect immunofluorescence reaction (RIFI) performed at the serology laboratory.

This study included all dogs diagnosed with VL, confirmed by the laboratory criteria, registered in urban areas by the local Zoonosis Control Division (ZCD). The serology techniques included enzyme-linked immunosorbent assays (ELISAs) and RIFI performed at the serology laboratory.

The Brazilian Ministry of Health recommends the demarcation of VL transmission areas in each municipality. These areas are stratified according to the average number of cases reported in the last three years. Municipalities with less than an average of 2.4 cases are classified as areas of sporadic transmission. Municipalities reporting between 2.4 and less than 4.4 cases and above or equal to 4.4 cases are considered areas of moderate and active transmission, respectively^9^.

All data necessary for the sectorization of the municipality and drawing of maps were made available by the ZCD and GEPI of the Department of Health Surveillance, Municipal Health Secretariat of the city of Governador Valadares. The municipality of Governador Valadares comprises 150 neighborhoods. Based on their proximity and similarities in socioeconomic and environmental characteristics, the neighborhoods were grouped into nine sectors (A to I). 

The sites of human and canine VL cases were organized according to streets, buildings, and neighborhoods, and linked to the municipal land registry office (LRO) through a unique identification number for each plot of land. These numbers were generated in the municipal register database using the *NetTerm* program. CTM facilitated the precise location of the plots to be geocoded and the production of elaborate thematic maps.

To map human and canine VL cases, the codes for buildings and public places were organized in Excel spreadsheets and georeferenced using the ArcGIS software (ESRI, Redlands, CA, USA) developed for visualization of Geographic Information Systems (GIS) data.

Statistical analyses were performed using Excel spreadsheets and MedCalc statistical software (MedCalc Software bvba, Ostend, Belgium) (https://www.medcalc.org/calc/).

The Pearson linear correlation coefficient was used to measure the following correlations: a) canine population and human VL cases; b) canine population and canine VL cases; c) canine and human VL cases.

The study was approved by the Ethics and Research Committee of the Universidade do Vale do Rio Doce, Governador Valadares, Brazil (protocol no. 396.413/2013).

The municipality of Governador Valadares consists of 150 neighborhoods, and these were grouped into nine sectors (A to I) based on their proximity and socioeconomic and environmental characteristics (Figure 1).

**FIGURE 1:**
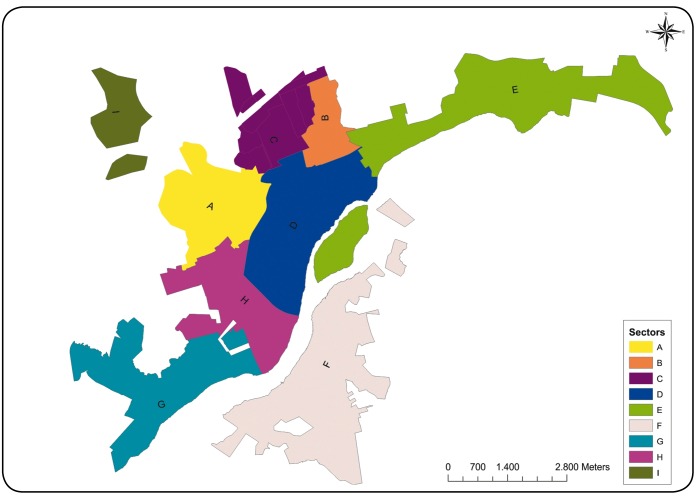
Distribution of neighborhoods in the municipality of Governador Valadares, Minas Gerais, by sectors (A to I).

GEPI reported 115 cases of human VL between 2008 and 2012 (Table 1). The first case occurred in 2008. There were 14, 30, 25, 21, and 25 cases reported in 2008, 2009, 2010, 2011, and 2012, respectively. The average number of VL cases during the last three years (2010 to 2012) was 23.6, well above 4.4; thus, the Brazilian Ministry of Health considers this region an active transmission area. Of the 115 cases, 17 patients died, with 3, 8, 0, 3 and 3 deaths reported in 2008, 2009, 2010, 2011 and 2012, respectively, corresponding to a mortality rate of 15.0% during this period. 

**TABLE 1:** Cases of canine and human visceral leishmaniasis and canine seroprevalence rate in Governador Valadares, Minas Gerais, by sector, from 2008 to 2012.

Sector	Dogs examined	Canine VL cases	Rate of seroprevalence	Human VL cases	Annual average of
	n	n	(%)	n	human cases of VL
A	5.717	1.830	32,0	40	8,0
B	3.168	1.112	35,0	21	4,2
C	1.944	649	33,3	5	1,0
D	3.939	1.363	34,6	19	3,8
E	2.621	667	25,4	4	0,8
F	5.062	1.289	25,4	9	1,8
G	3.037	674	22,1	2	0,4
H	3.174	754	23,7	12	2,4
I	1.062	284	26,7	3	0,6
**Total**	**29.724**	**8.622**	**29,0**	**115**	

From 2008 to 2012, a total of 29,724 samples were collected from dogs. Of the collected samples, 8,622 were seropositive for canine VL, with 1,481 seropositive (26.5%) in 2008, 1,557 (24.5%) in 2009, 1,087 (27.2%) in 2010, 1,759 (34.2%) in 2011, and 2,738 (32.0%) in 2012.

Table 1 includes canine and human VL cases from 2008 to 2012, showing the relationship between canine seroprevalence and human VL cases according to municipality sectors.

Figure 2 shows the superposition of the spatial distribution of human and canine VL cases, indicating large numbers of human cases in sectors with high concentrations of canine VL cases. 

**FIGURE 2:**
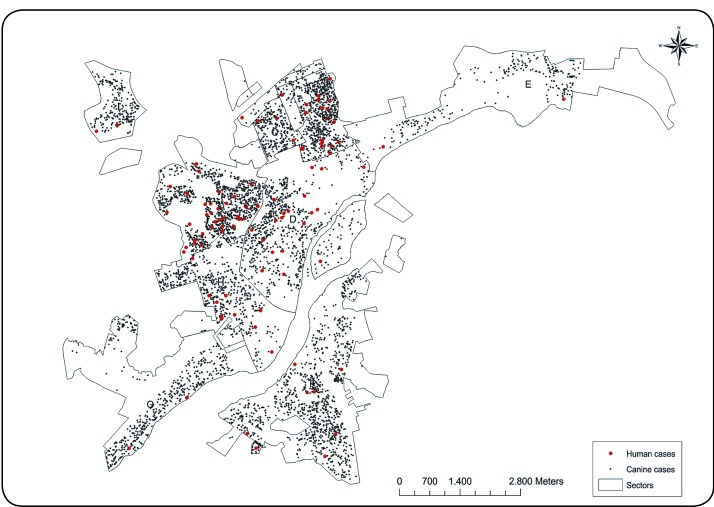
Spatial distribution of human and canine cases of visceral leishmaniasis in Governador Valadares, Minas Gerais, from 2008 to 2012.

Correlation analyses showed that a correlation exists between the canine population and canine VL cases (0.9918) and also between canine and human VL cases (0.973), significant at 0.1%. 

The number of dogs in the municipality did not correlate with a higher incidence of human VL cases. However, the highest number of canine VL cases correlated with a higher incidence of human VL cases.

In an analysis conducted between 2008 and 2011, Barata et al. demonstrated a trend of increased prevalence of VL canine cases in certain neighborhoods of the municipality of Governador Valadares, presenting a high density of *L. longipalpis* ^7^. The present study, conducted between 2008 and 2012, reported that the correlation between the canine population and number of canine VL cases was highly positive (0.9918), indicating that higher the number of dogs, the greater the probability of infected dogs. The first human case was reported in 2008 in an urban area in the municipality of Governador Valadares, leading to an epidemic outbreak^7^. Between 2008 and 2012, 115 cases were reported. Similar data were reported for municipalities located in the northern region of MG, with 119 cases in five years in the municipality of Janaúba and 97 cases in three years in Montes Claros^10^. Of the 115 cases reported in the municipality of Governador Valadares between 2008 and 2012, 17 patients died, presenting a 15.0% mortality rate. This rate was significantly higher than the national and MG averages (6.9% and 10.3%, respectively) during the period of 2008-2012^11^.

The reported canine seroprevalence was 5.8% between 2008 and 2009 in the municipality of Montes Claros^10^. This rate was lower than that reported in Governador Valadares, which was 25.5% during the same period (data not shown). In 2015, the seroprevalence was reportedly 14.8% in the municipality of Ipatinga, about 105 km from Governador Valadares^12^. Between 2008 and 2012, the canine seroprevalence rate in Governador Valadares was 29.0%.

Using spatial analysis, Oliveira et al. (2001) presented strong evidence that cases of human VL from 1994 to 1997 occurred in areas of the city of Belo Horizonte where the canine prevalence rate was high^13^. Similarly, Borges et al. (2009) demonstrated a correlation between human and canine VL cases in Belo Horizonte, the capital of the state of MG^14^. The current study demonstrated a positive correlation between cases of human and canine VL in the municipality of Governador Valadares.

A limitation of this study is the absence of a statistical analysis demonstrating the sector-wise correlation between human and canine cases.

The sectorization of the municipality of Governador Valadares in the present study allowed a better visualization of the disease distribution. All sectors had a high prevalence of canine VL cases. However, in sectors with a higher incidence of human cases (A, B and D), rates of canine seroprevalence were even higher. Sectorization by neighborhoods has also been conducted in other municipalities, such as Janaúba, MG, which is considered an area of intense VL transmission^15^.

Therefore, the results of this study indicated that dogs play an important role in the transmission of human VL. Furthermore, a reassessment of the disease control strategies, using the epidemiological surveillance of the municipality, would be of immense interest.
